# Supplementary material for: Cellulose/Zeolitic Imidazolate Framework (ZIF-8) Composites with Antibacterial Properties for the Management of Wound Infections
Source: J Funct Biomater. 2023 Sep 13;14(9):472. doi: 10.3390/jfb14090472 (PMC10532010; doi:10.3390/jfb14090472)
Supplement: Supplementary file 1 [file jfb-14-00472-s001.zip › jfb-2539054-supplementary.pdf]

Journal of *Functional Biomaterials*

Article

Cellulose/Zeolitic Imidazolate Framework (ZIF-8) Composites with Antibacterial Properties for the Management of Wound Infections

Valentina Di Matteo [1], Maria Francesca Di Filippo [2], Barbara Ballarin [1,3,4], Giovanna Angela Gentilomi [5,6], Francesca Bonvicini [5,*], Silvia Panzavolta [2,4] and Maria Cristina Cassani [1,7,*]

1 Department of Industrial Chemistry "Toso Montanari", University of Bologna, Viale del Risorgimento 4, 40136 Bologna, Italy; valentina.dimatteo5@unibo.it (V.D.M.); barbara.ballarin@unibo.it (B.B.)
2 Department of Chemistry "G. Ciamician", University of Bologna, Via Selmi 2, 40126 Bologna, Italy; maria.difilippo5@unibo.it (M.F.D.F.); silvia.panzavolta@unibo.it (S.P.)
3 Center for Industrial Research—Fonti Rinnovabili, Ambiente, Mare e Energia CIRI FRAME, University of Bologna, Viale del Risorgimento 2, 40136 Bologna, Italy
4 Center for Industrial Research—Advanced Applications in Mechanical Engineering and Materials Technology CIRI MAM, University of Bologna, Viale del Risorgimento 2, 40136 Bologna, Italy;
5 Department of Pharmacy and Biotechnology, University of Bologna, Via Massarenti 9, 40138, Italy; giovanna.gentilomi@unibo.it (G.A.G.)
6 Microbiology Unit, IRCCS Azienda Ospedaliero-Universitaria di Bologna, Via Massarenti 9, 40138 Bologna, Italy
7 Health Sciences and Technologies—Interdepartmental Center for Industrial Research (HST–ICIR), Alma Mater Studiorum—University of Bologna, 40064 Ozzano dell'Emilia, Bologna, Italy
* Correspondence: francesca.bonvicini4@unibo.it (F.B.); maria.cassani@unibo.it (M.C.C.)

Citation: Di Matteo, V.; Di Filippo, M.F.; Ballarin, B.; Gentilomi, G.A.; Bonvicini, F.; Panzavolta, S.; Cassani, M.C. Cellulose/Zeolitic Imidazolate Framework (ZIF-8) Composites with Antibacterial Properties for the Management of Wound Infections. *J. Funct. Biomater.* **2023**, *14*, x. https://doi.org/10.3390/xxxxx

Academic Editor: Aharon Azagury, Malka Shilo

Received: 18 July 2023
Revised: 29 August 2023
Accepted: 8 September 2023
Published: 13 September 2023

Copyright: © 2023 by the authors. Submitted for possible open access publication under the terms and conditions of the Creative Commons Attribution (CC BY) license (https://creativecommons.org/licenses/by/4.0/).

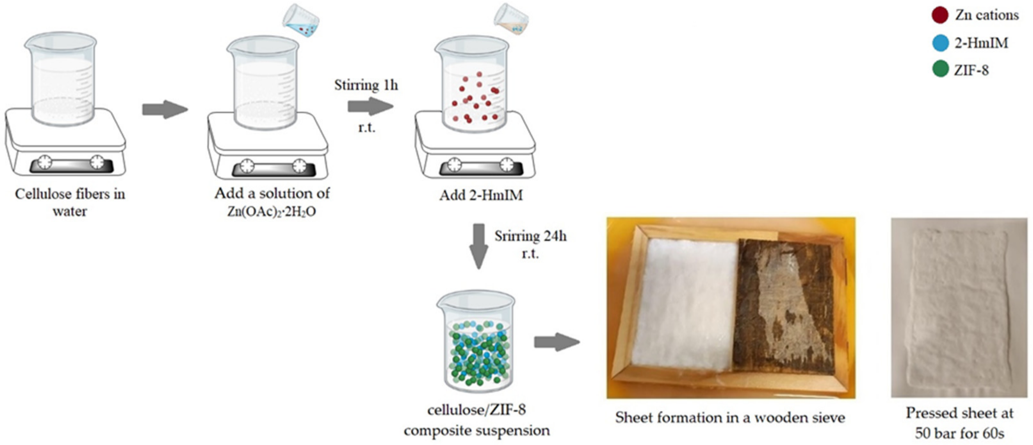

Figure S1. Schematic representation (done with "BioRender") of the Cell@ZIF-8 samples synthesis.

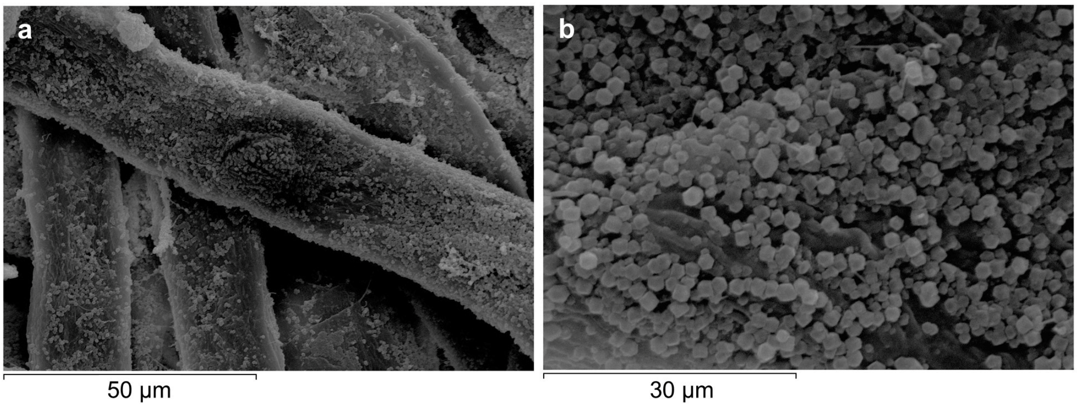

Figure S2. SEM image of the *activated* Cell2@ZIF-8 sample at different scales: a) 50 and b) 30 μm).

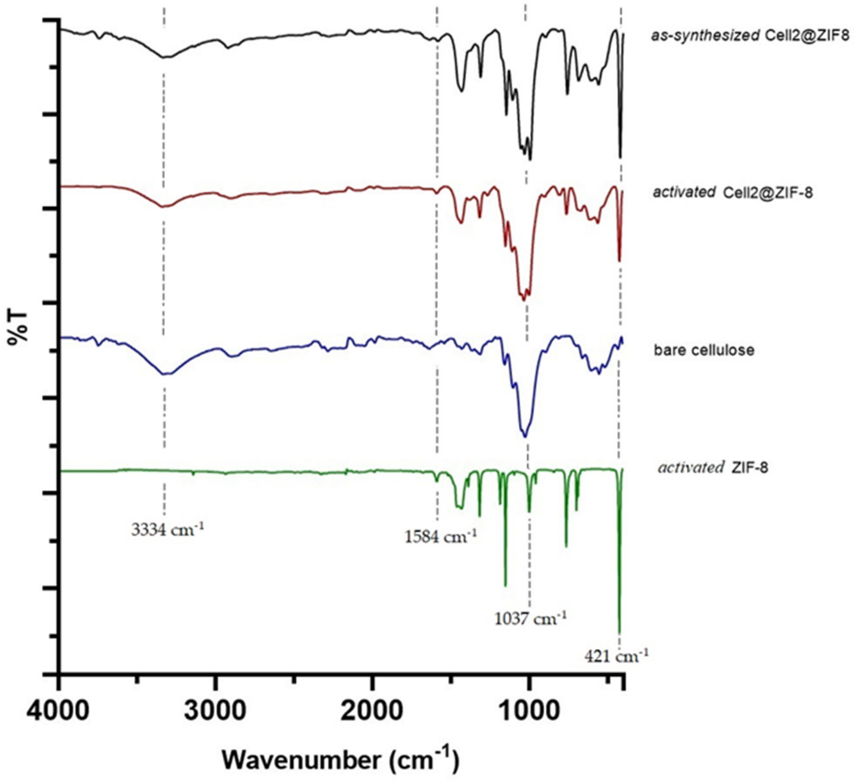

Figure S3. Comparison between the IR spectra of the bare cellulose, Cell2@ZIF-8 and pure ZIF-8 samples.

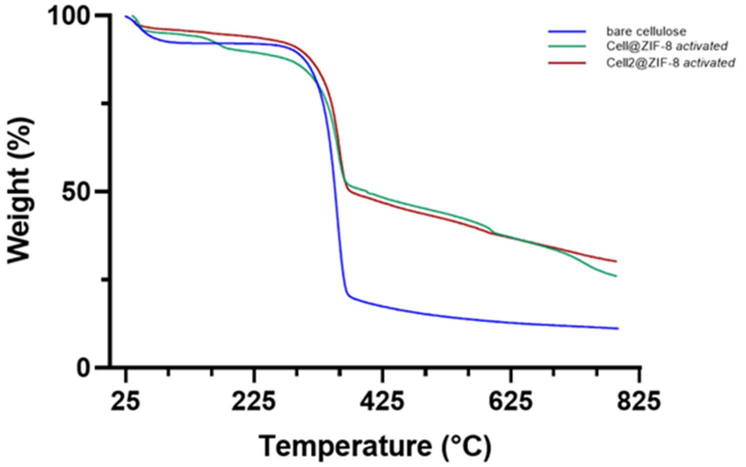

Figure S4. Comparison between the thermograms of bare cellulose (blue), *activated* Cell@ZIF-8 (green) and *activated* Cell2@ZIF-8 (red) samples.

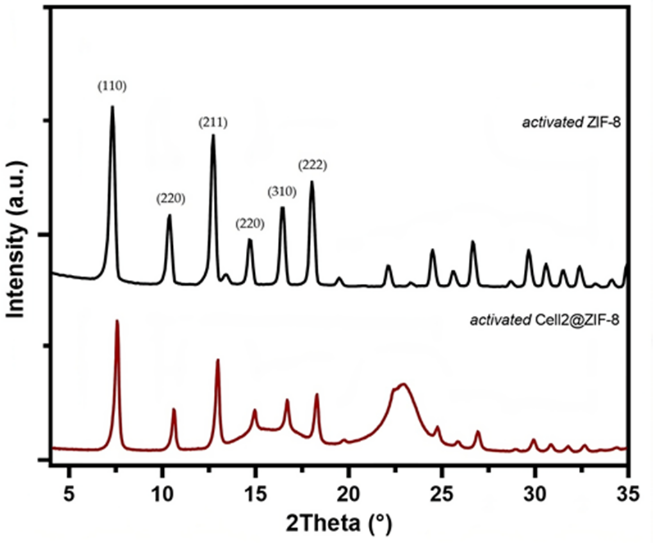

Figure S5. XRD diffractograms of *activated* Cell2@ZIF-8 (red) and pure ZIF-8 (black).

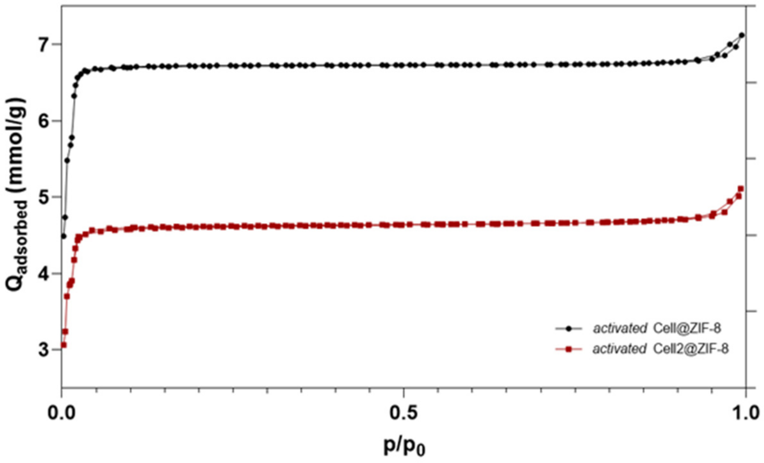

Figure S6. Absorption and desorption isotherms of *activated* Cell@ZIF-8 (black) and *activated* Cell2@ZIF-8 (red) samples.

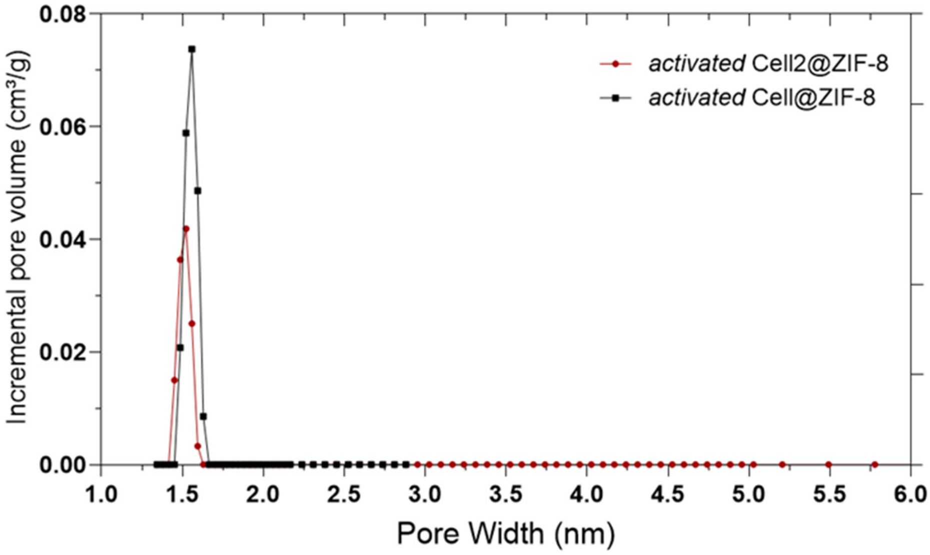

Figure S7. Incremental Pore Volume distribution obtained by using DFT of *activated* Cell@ZIF-8 and *activated* Cell2@ZIF-8 samples. The integral of these curves is the total pore volume reported in Table 2.

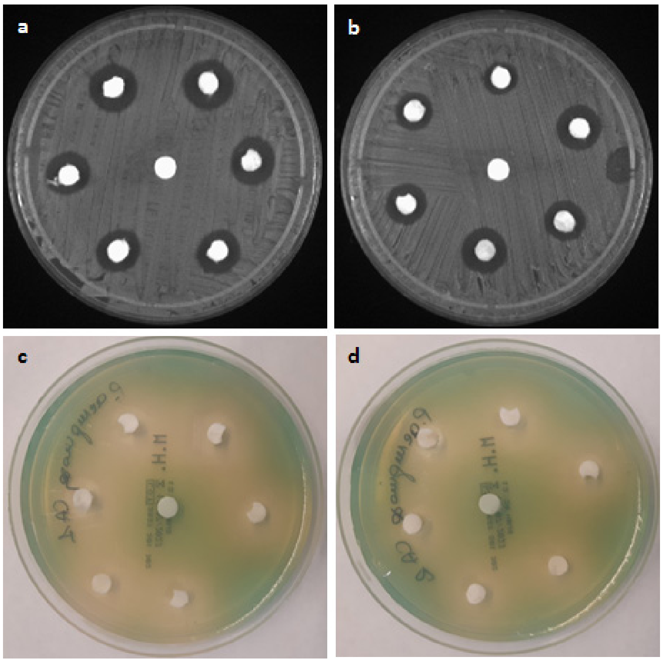

Figure S8. Representative pictures of the disk-diffusiont tests on *S. epidermidis* with *activated* Cell@ZIF-8 (**a**) and Cell2@ZIF-8 samples (**b**) and on *P. aeruginosa* with *activated* Cell@ZIF-8 (**c**) and Cell2@ZIF-8 samples (**d**). Six replicates of each sample and a cellulose bare sample (in the middle of the plate) are tested on the same bacterial suspension to measure the intra-assay variability of the materials.

Table S1. Antibacterial activity determined with the standardized disk-diffusiont test. The diameters of the inhibition zone are expressed in millimeters (mm).

Reference strains	Bare cellulose	Cell@ZIF-8 *as-synthetized*	Sterile paper disk	GNT 10 μg	VNC 10 μg
S. aureus ATCC 25923	NA[1]	9 ± 1	NA	18 ± 1	14 ± 1
S. epidermidis ATCC 12228	NA	15 ± 1	NA	22 ± 1	15 ± 1
E. coli ATCC 25922	NA	8 ± 1	NA	19 ± 1	NA
P. aeruginosa ATCC 27853	NA	13 ± 1	NA	18 ± 1	NA

[1]NA: Not appeared

Table S2. Antibacterial activity determined with the agar-cup method. The diameters of the inhibition zone are expressed in millimeters (mm).

Reference strains	*S. epidermidis*	*P. aeruginosa*
undiluted ZIF-8 powder *as-synthetized*	19 ± 1	20± 1
ten-fold diluted ZIF-8 powder *as-synthetized*	13 ± 1	13 ± 1
undiluted ZIF-8 powder *activated*	19 ± 1	20± 1
ten-fold diluted ZIF-8 powder *activated*	13 ± 1	13 ± 1
